# Evaluation of a novel dye for near‐infrared fluorescence delineation of the ureters during laparoscopy

**DOI:** 10.1002/bjs5.59

**Published:** 2018-04-19

**Authors:** M. Al‐Taher, J. van den Bos, R. M. Schols, B. Kubat, N. D. Bouvy, L. P. S. Stassen

**Affiliations:** ^1^ Department of Surgery Maastricht University Medical Centre Maastricht The Netherlands; ^2^ Department of Plastic, Reconstructive and Hand Surgery Maastricht University Medical Centre Maastricht The Netherlands; ^3^ Department of Pathology Maastricht University Medical Centre Maastricht The Netherlands

## Abstract

**Background:**

Iatrogenic ureteric injury remains a risk in laparoscopic pelvic procedures. Near‐infrared fluorescence (NIRF) imaging is a promising new technique for enhanced intraoperative visualization of anatomical structures that could improve the safety of laparoscopic surgery. A new dye, IRDye^®^ 800‐BK, has been developed for intraoperative visualization of the ureters using NIRF. The present study was a first evaluation of the performance of IRDye^®^ 800‐BK for ureteric imaging during NIRF laparoscopy.

**Methods:**

This study consisted of three parts: real‐time in vivo NIRF imaging using IRDye^®^ 800‐BK in pigs during laparoscopic surgery, ex vivo NIRF imaging of freshly explanted pig ureters and ex vivo NIRF imaging of explanted human ureters.

**Results:**

In all animals, both left and right ureters were visualized throughout the laparoscopic procedure for 120 min, with the best results at a dose of 0·15 mg dye per kg bodyweight. NIRF imaging was successful in all human and porcine ureters studied, with a range of dye concentrations.

**Conclusion:**

NIRF imaging of the ureters using IRDye^®^ 800‐BK was used successfully both in vivo in a porcine model, and ex vivo in porcine and human ureters.

## Introduction

Despite advances in laparoscopic surgery, iatrogenic ureteric injuries during laparoscopy may still occur^1^. Several studies^2^, ^3^, ^4^, ^5^ have reported an incidence of between 0·1 and 7·6 per cent in colorectal and gynaecological surgery, with more than 80 per cent of injuries going unrecognized during surgery. Ureteric injury leads to significant postoperative morbidity even if identified and repaired during the same procedure^6^. Near‐infrared fluorescence (NIRF) imaging is a promising new technique for easier and earlier intraoperative visualization of target organs with the potential to improve the safety of laparoscopic surgery^7^ ^8^. Indocyanine green (ICG) and methylene blue are currently the only clinically available dyes that may be helpful in intraoperative visualization of the ureter^9^, ^10^, ^11^. However, a feasibility study^12^ recently showed that, even though imaging using methylene blue was safe and feasible, it did not provide a practical advantage over conventional laparoscopic imaging for identification of the ureter during laparoscopic colorectal surgery.

IRDye^®^ 800CW (LI‐COR Biosciences, Lincoln, Nebraska, USA) is an experimental dye that allows intraoperative visualization of crucial anatomical structures using NIRF imaging. Animal studies^13^, ^14^, ^15^ have successfully demonstrated the potential of this dye for identification of the ureters. However, a major disadvantage of IRDye^®^ 800CW is its cost, which is almost tenfold that of ICG.

Recently a new preclinical dye, IRDye^®^ 800‐BK (LI‐COR Biosciences), has been developed specifically for intraoperative NIRF visualization of the ureters. Because of its hydrophilic nature, it is primarily cleared by the kidneys and may therefore have great potential for real‐time NIRF imaging of the ureter. According to the manufacturer, the price of IRDye^®^ 800‐BK is expected to be similar to that of ICG. The aim of the present study was to evaluate this dye for ureteric imaging during NIRF laparoscopy.

## Methods

This study was conducted at the central animal facilities of Maastricht University (Maastricht, The Netherlands). Animals were used in compliance with the regulations of Dutch legislation for animal research and following a protocol approved by the local animal ethics committee. Three female Dutch Landrace pigs were used as well as explanted porcine and human ureters. The ARRIVE guidelines^16^ were followed for reporting these experiments.

A laparoscopic fluorescence imaging system (Karl Storz, Tuttlingen, Germany) was used. The D‐Light P system includes a plasma light guide and a 30° 10‐mm laparoscope applicable for white light and NIRF imaging. It enables excitation of the dye under evaluation. A foot pedal allows the surgeon to switch easily between the two imaging modalities. All procedures were recorded digitally.

IRDye^®^ 800‐BK has a maximum absorption at 774 nm and a maximum emission at 790 nm. The dye was prepared before use according to the manufacturer's instructions and diluted in sterile phosphate‐buffered saline (PBS) to a concentration of 1 mg/ml.

The experiments consisted of three parts. The performance of the dye was tested *in vivo* in a pig model, as a dose‐finding study. Then, the influence of the thickness of the ureter wall and the dose administered on the signal achieved was tested during *ex vivo* experiments using both porcine and human ureters.

### In vivo fluorescence imaging

The dye was tested during laparoscopic surgery in three female Landrace pigs, each weighing between 39 and 39·6 kg. Premedication consisted of intramuscular injection of azaperone 3 mg/kg, ketamine 10 mg/kg and atropine 0·05 mg/kg. Anaesthesia was induced with intravenous thiopental 10–15 mg/kg. After intubation, the pigs were maintained under anaesthesia with isoflurane and oxygen. Vital parameters were monitored continuously.

The pigs were placed supine in a steep Trendelenburg position and three trocars were introduced: a 12‐mm trocar in the midline superior to the umbilicus and two 5‐mm trocars on either side. Pneumoperitoneum was established with carbon dioxide. Surgery and NIRF imaging were performed by two surgeons specifically experienced in laparoscopic surgery and NIRF imaging. The ureters were not dissected or exposed in any animal.

The first pig received an intravenous dose of 6 mg (6 ml) IRDye^®^ 800‐BK as a bolus, which was in the range of doses used in previous experiments with IRDye^®^ 800CW. The second and third pigs received half (3 mg) and double (12 mg) this dose respectively. The dye was administered directly after introduction of the laparoscopic trocars. Observation of the left and right ureters, in white light and fluorescence mode, was planned for every 10 min for 1 h, starting after 20 min, and every 20 min during the second hour. However, the ureter was visualized clearly after 20 min in the first pig, so it was decided to initiate visualization right from the start of the operation in the next two animals. After surgery, the pigs were killed in accordance with the guidelines of the Dutch legislation for animal research.

### Ex vivo fluorescence imaging of porcine ureters

Freshly explanted pig ureters were collected from the abattoir on the day of the experiments. Ureters were flushed with PBS followed by flushing with the diluted IRDye^®^ 800‐BK, during which first the distal and then the proximal part of the ureter was clamped, ensuring watertight closure on both sides. The whole process was recorded with the laparoscope in fluorescence mode at a distance of 10 cm from the ureter. The same background was used in all ureters studied. The following dilutions were examined: 1 : 4, 1 : 16, 1 : 64 and 1 : 256.

### Ex vivo fluorescence imaging of human ureters

Tissue was obtained from the Maastricht Pathology Tissue Collection. Collection, storage, and use of tissue and patient data were in agreement with the code for proper secondary use of human tissue in the Netherlands (http://www.federa.org). A pathologist explanted the ureters during post‐mortem examination of patients not previously known to have urological diseases or ureteric abnormalities. All ureters were explanted within 2 weeks before experimentation and stored at –20°C. They were defrosted at room temperature 3 h before the experiments. The NIRF imaging procedure was similar to that for *ex vivo* porcine ureters. Dilutions examined were: 1 : 1, 1 : 4, 1 : 16, 1 : 64, 1 : 256, 1 : 1024 and 1 : 4096.

### Measurement of ureter wall thickness

In *ex vivo* experiments, a representative cross‐section of the distal third of the ureter was analysed microscopically, on the basis that this part of the ureter is usually of interest during laparoscopic colorectal and gynaecological surgery^17^. Ureter wall thickness was defined as the distance from the surface of the luminal urothelium to the outer layer of smooth muscle. Wall thickness values presented are the mean of nine measurements per cross‐section.

### Analysis

Video recordings of the laparoscopic procedures were assessed for the degree of fluorescence illumination using OSIRIX version 5.0.1 imaging software (Pixmeo, Geneva, Switzerland). Signal and target‐to‐background ratio (TBR) were determined from representative screenshots. The TBR was defined as the mean fluorescence intensity (FI) of three points of interest in the target (ureter) minus the mean FI of three points of interest in the background directly adjacent to the ureter, divided by the mean FI of three points of interest in the background^18^.

The *ex vivo* experiments were performed in a completely darkened room with the laparoscope 10 cm from the explanted ureter. The fluorescence from an area 1 cm lateral to the ureter was chosen as the background fluorescence for each ureter.

## Results

### In vivo fluorescence imaging

Pig 1, with a bodyweight of 39·4 kg, received an intravenous bolus of 6 mg dye, resulting in a dose of 0·15 mg dye per kg bodyweight. The first clear and distinct visualization of both the left and right ureters in fluorescence mode occurred 20 min after administration of the dye. Pig 2, with a bodyweight of 39·0 kg, received an intravenous bolus of 3 mg dye (0·08 mg dye per kg bodyweight). The first clear and distinct visualization of both ureters in fluorescence mode occurred 20 min after dye administration. Pig 3, with a bodyweight of 39·6 kg, received an intravenous bolus of 12 mg dye (0·30 mg per kg bodyweight). The first visualization of both ureters in fluorescence mode occurred 1 min after administration of dye. At this stage, it was the wall of both ureters that became clearly visible. The first peristaltic movements of urine through the ureter were observed 10 min after administration of dye.

**Figure 1:**
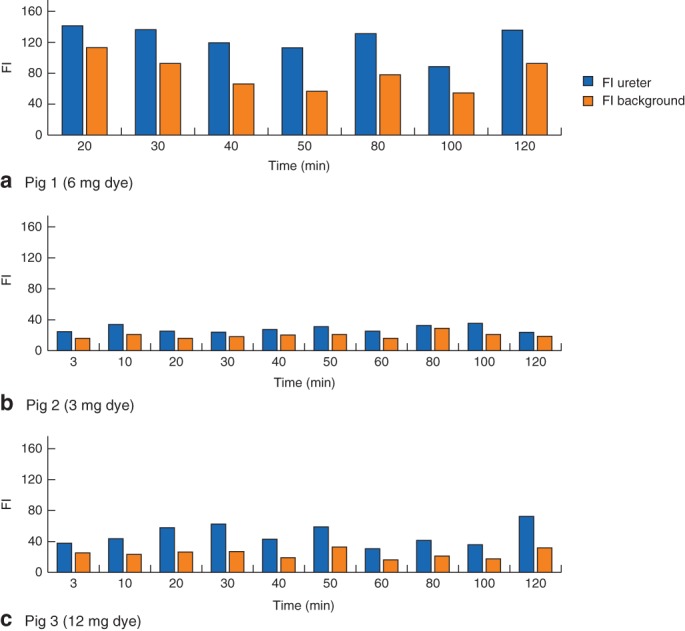
Absolute fluorescence intensity (FI) at various time points for the left ureter in **a** pig 1 (IRDye^®^ 800‐BK dose 6 mg), **b** pig 2 (dose 3 mg) and **c** pig 3 (dose 12 mg), showing the mean FI for the target (ureter) and its background

**Figure 2:**
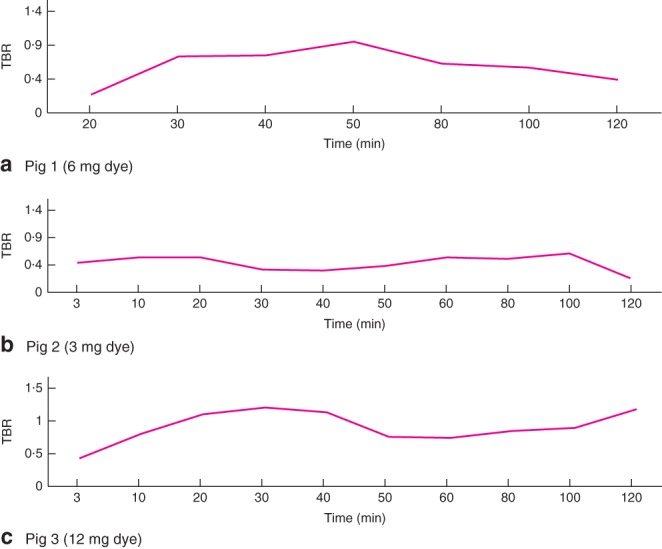
Target‐to‐background ratio (TBR) at various time points during laparoscopy for the left ureter in **a** pig 1 (IRDye^®^ 800‐BK dose 6 mg), **b** pig 2 (dose 3 mg) and **c** pig 3 (dose 12 mg)

In all three pigs, both the left and right ureter were visualized with peristaltic movement until the end of the experiment after 120 min, confirming transportation of the dye‐containing urine through the ureter. A very clear and certain course of the ureter could be observed subjectively in pigs 1 and 3. In contrast, the FI of the ureter in pig 2 was significantly lower than in the other two pigs, making it difficult to distinguish the ureter from the surrounding tissues. The measured FI is shown in *Fig*. 1. Pig 1 had the highest absolute FI measured, followed by pig 3 and pig 2. *Fig*. 2 shows TBRs at various points during the laparoscopy for the left ureter in the three pigs. The TBR was highest for the highest doses administered.

**Figure 3:**
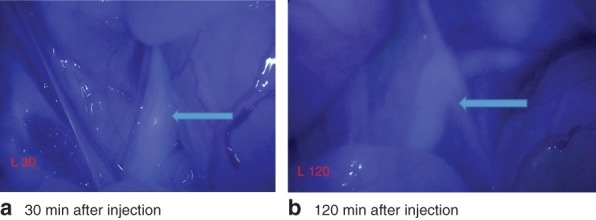
Screenshots of left ureter **a** 30 min and **b** 120 min after injection of 6 mg IRDye^®^ 800‐BK in pig 1. Arrow indicates the ureter

In all pigs, the subjective maximum intensity of the fluorescence signal was seen at moments of peristaltic contractions of the ureter, showing transport of intraluminal urine from proximal to distal. Screenshots of the left ureter 30 and 120 min after injection of 6 mg IRDye^®^ 800‐BK in pig 1 are shown in *Fig*. 3, with the ureter distinct from the surrounding tissue.

### Ex vivo fluorescence imaging of porcine ureters

**Figure 4:**
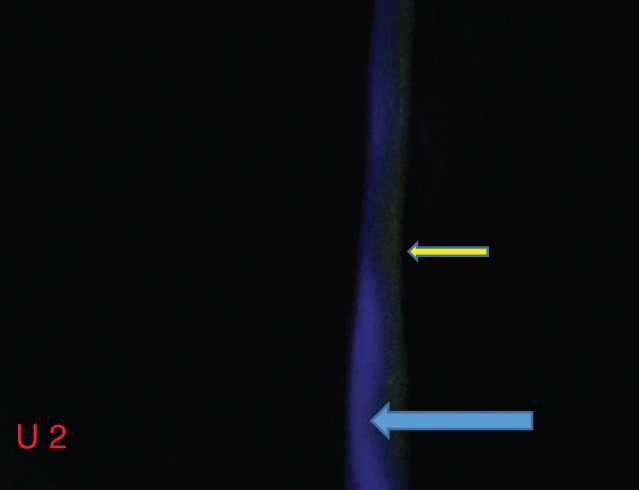
Screenshot of explanted pig ureter 2. The blue arrow indicates the ureter, and the yellow arrow the overlying fatty tissue

The wall thickness of the four pig ureters ranged from 93·3 to 129·0 (mean 113·6) μm. A clear delineation of all four ureters was possible using fluorescence imaging, even at the lowest concentration of dye tested. FI and TBR ranged from 38 to 56 and 6·60 to 10·20 respectively (*Table* 1). An initial lowering of the concentration seemed to improve the signal, despite increasing wall thickness (TBR ureter 2 *versus* 1). Further dilution gave varying results: an initial decrease in the signal, as observed in ureter 3, but an increase in ureter 4. Variations in concentration and wall thickness are shown in *Table* 1. A further observation was that fatty tissue on the ureter strongly influenced the fluorescence signal (*Fig*. 4). A signal could be obtained only in areas where the ureter was not covered by fat as seen by the naked eye. This effect was consistent across all ureters and at all concentrations tested.

**Table 1:** Influence of wall thickness and dye concentration on fluorescence intensity and target‐to‐background ratio of pig ureters

Ureter no.	Concentration of IRDye^®^ 800‐BK in PBS	Ureter wall thickness (μm)	Fluorescence intensity	Target‐to‐background ratio
1	1 : 4	119·2	38	6·60
2	1 : 16	129·0 (108)	56	10·20
3	1 : 64	112·7 (95)	40	7·00
4	1 : 256	93·3 (78)	46	8·20

Values in parentheses are percentage of thickness of ureter 1. PBS, phosphate‐buffered saline.

**Figure 5:**
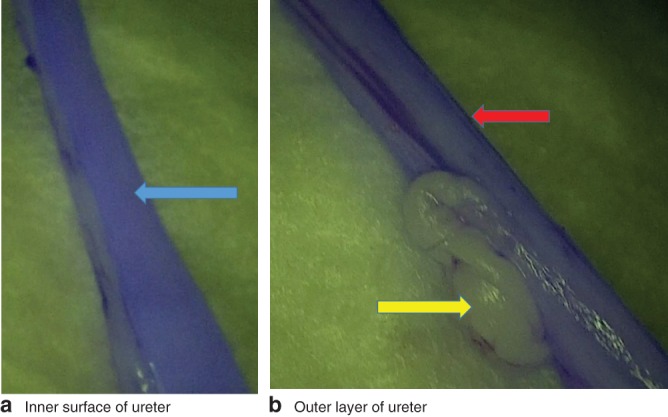
Screenshots of explanted pig ureter 1: **a** inner surface of the ureter and **b** outer layer of the same ureter. The blue arrow indicates the luminal surface of the ureter, the red arrow the outer layer of the ureter, and the yellow arrow the overlying fatty tissue

After rinsing the ureter extensively with pure PBS, a low fluorescence signal remained in the ureter, both in the lumen and in the outer layer of the ureter (Fig. 5), suggesting uptake of the dye in the wall of the ureter.

### Ex vivo fluorescence imaging of human ureters

Wall thickness of seven post‐mortem human ureters ranged from 133·8 to 189·9 (mean 162) μm. Clear delineation of all seven ureters was possible using fluorescence imaging, at all dye concentrations, with FI and TBR ranging from 11 to 49 and 1·20 to 8·80 respectively (*Table* 2). Again, areas of ureter covered by a layer of fatty tissue did not show fluorescence.

**Table 2:** Influence of wall thickness and concentration of dye on fluorescence intensity and target‐to‐background ratio of human ureters

Ureter no.	Concentration of IRDye^®^ 800‐BK in PBS	Ureter wall thickness (μm)	Fluorescence intensity	Target‐to‐background ratio
1	1 : 1	150·8	42	7·40
2	1 : 4	171·6 (114)	49	8·80
3	1 : 16	158·9 (105)	37	6·40
4	1 : 64	167·8 (111)	45	8·00
5	1 : 256	189·9 (126)	25	4·00
6	1 : 1024	133·8 (89)	22	3·40
7	1 : 4096	162·1 (107)	11	1·20

Values in parentheses are percentage of thickness of ureter 1. PBS, phosphate‐buffered saline.

## Discussion

IRDye^®^ 800‐BK has been developed specifically for intraoperative NIRF visualization of the ureters. The goals of the present experiments were to see whether the new dye would be suitable for visualization of the ureter and to perform a first preliminary evaluation of factors likely to influence the signal produced.

The study confirmed visualization of the ureters in real time both *in vivo* and *ex vivo*. *In vivo*, administration of a bolus of 6 and 12 mg (0·15 and 0·30 mg/kg respectively) allowed excellent and clear visualization of the ureters in NIRF mode. The dose of 3 mg (0·08 mg/kg) also provided enhanced imaging of the ureter, although the FI was weak.

The maximum intensity of the fluorescence signal was seen during contractions of the ureter, showing the transport of intraluminal urine from proximal to distal. The relationship with peristalsis is a slight drawback, as the signal is not present permanently, although this will be the case for every signal produced by a dye transported in urine. This drawback seems greatly surpassed by the excellent signal during these episodes of contractions.

A signal was observed for up to 120 min after the administration of dye, when the surgical procedures were terminated. A study with longer continuous NIRF visualization of the ureter may be beneficial.

Each ureter showed varying FI and TBR throughout the *in vivo* study. A possible explanation may have been failure to standardize the laparoscope–target distance. It is possible that the laparoscope was at different distances from the ureter, resulting in different amounts of fluorescence being detected and influencing the TBR. Kono and colleagues^19^ reported that the signal contrast on fluorescence images of bile duct samples differed significantly between the laparoscopic imaging systems, and tended to decrease as the laparoscope–target distance increased and porcine tissues covering the samples became thicker.

In the *in vivo* study, all concentrations of dye tested resulted in enhanced visualization of the ureter compared with conventional white light, as indicated by a positive TBR value. Doses of 6 and 12 mg gave the best intraoperative results. Doses that resulted in a subjectively better signal and a higher measured absolute intensity did not always result in a higher TBR. This may be explained by the influence of the dose both on target and background signals. A low FI of the target may be accompanied by a very low FI of the background, whereas a higher FI of the target may be accompanied by a high background signal.

At all doses studied it was observed that urine flow in the ureter was not continuous but related to contractions, with the fluorescent signal related to urine flow, resulting in fluctuations in the fluorescence signal detected. As a result, the observed and measured signals and TBRs may vary over time during *in vivo* studies. In contrast, in the *ex vivo* experiments a uniform background could be chosen, which enabled comparison of the TBR between experiments.

To further study the dose–visibility relationship, explanted pig ureters and dye dilutions ranging from 1 : 4 to 1 : 256 were used. A clear delineation of the ureters was obtained at all doses, with a TBR ranging from 6·6 to 10·20. As it has been suggested previously that ureter wall thickness may have a negative influence on NIRF intensity^12^, and that findings in animal studies may not be representative of humans, human ureters with their thicker walls were also subjected to *ex vivo* NIRF imaging. Dye concentrations from 1 : 1 up to 1 : 4096 showed successful NIRF imaging in all human ureters, with TBRs ranging from 1·20 to 8·80. The increased wall thickness therefore did not prevent the fluorescence signal, although a decrease in FI was observed at the lowest concentrations. In both the porcine and human *ex vivo* experiments, not all results for TBR had a logical explanation, but it seems that the concentration of dye had a greater influence than thickness of the ureter wall.

No complications or adverse reactions attributable to the dye were observed during any operations. Only a slight and transient decrease in intraoperative peripheral oxygen saturation was noted during the first minute after injection of the fluorescent dyes.

A limitation of the *ex vivo* experiments is that two influences were studied at the same time: dye concentration and wall thickness. Ideally, different concentrations of dye should be tested in the same ureters, and the same concentration in different ureters. The first of these types of experiment is not feasible, because the fluorescent signal was retained in the ureter wall. Such an experiment can therefore be performed only using artificial material. The second experiment has not yet been undertaken.

Despite the promising results, the present findings must be interpreted with caution. Owing to the limited availability of pigs and ureters, it was not possible to study enough ureters for solid statistical conclusions to be drawn. Another limitation is that concentrations of dye in the urine were not measured. This requires availability of the structural formula of the dye and a method for such measurement. These were not available to the authors in this early phase of development and application of the dye.

It was disappointing in the *ex vivo* study that even the smallest layer of fat covering the ureter prevented a decent signal from being obtained. It is known that penetration of the NIRF signal is limited to approximately 10 mm^20^. NIRF imaging with a stronger dye or with optimized equipment was hoped to enhance such penetration. The present experiment illustrates that the signal may be improved with use of a better dye, but that this does not affect depth penetration *per se*. In the *in vivo* study, nevertheless, the porcine ureters could be identified clearly without any manipulation or dissection of the overlying tissues. This suggests that IRDye^®^ 800‐BK has the potential to detect the ureter in spite of the overlying peritoneum. Future studies should evaluate the maximum depth of penetration of the NIRF signal and the clinical value of this dye in human subjects.

This novel dye enables visualization of the ureters. NIRF imaging with this dye seems a valuable addition to conventional white light laparoscopy. Further studies are needed to see if it can become a worthwhile addition to improve clinical practice.
